# Exploring educational impacts among pre, during and post COVID-19 lockdowns from students with different personality traits

**DOI:** 10.1186/s41239-023-00388-4

**Published:** 2023-04-03

**Authors:** Yong Zheng, Shuaiqi Zheng

**Affiliations:** Illinois Institute of Technology, Chicago, IL USA

**Keywords:** Personality traits, COVID-19, Pandemic, Lockdown, Academic performance

## Abstract

The influence of personality traits on educational outcomes has been widely recognized and studied. Research has explored its effects on factors such as student satisfaction, academic anxiety, and dishonesty, particularly during the COVID-19 pandemic. However, there has been a lack of studies comparing the learning behaviors and performance of students with different personality traits during the pre, during, and post-COVID-19 lockdown periods. This study fills this gap by analyzing the differences in academic metrics, such as class grades and assignment submissions, among students with varying personality traits during different lockdown periods. Our research, based on a dataset of 282 graduate students in the USA, identified correlations and patterns between lockdown periods, personality traits, and academic metrics. For example, the class grades and the rate of late submissions were affected by different lockdown periods. Students with lower degree in extraversion and agreeableness made less attempts in assignment submissions. These findings can assist educators in identifying impacted students and developing effective teaching strategies at early stage in future incidents.

## Introduction

The COVID-19 pandemic has affected many aspects of life, including education, and has led to adaptations in the way learning is carried out. During lockdowns, schools and students had to adapt to remote learning. The shift to remote learning during the COVID-19 pandemic has brought about several new challenges that have affected students, teachers, and parents. These challenges may include but not limited to: access to Internet and technologies for remote learning (Lockee, 2021; Mbhiza, 2021), difficulties in learning engagement (Salas-Pilco, 2022, Tamah et al., 2020), inequities in education (Blaskó, 2022; Walters, 2020), increased academic anxiety (Hong, 2021; Kamran & Naeim, 2021; Rashid, 2022), mental health (Maqsood, 2021; Zhai & Du, 2020), and so forth.

Several studies have been conducted to understand the impact of the COVID-19 pandemic on learning and to compare remote learning with traditional face-to-face academic learning. The results of these studies may vary according to geographical regions and universities. For example, Pereira and Guerreiro (2021) discovered a significant raise in usage of online learning resources in a Portuguese university during the first wave of COVID-19 and an increase afterwards. Albeta et al. found no significant differences in learning performance between pre- and during-lockdown periods in Indonesia (Albeta, 2021). Feng et al. reported a decline in learning performance due to the COVID-19 pandemic in China (Feng, 2021). Karadag et al. discovered that universities with higher distance education capacities got higher satisfaction scores in Turkey (Karadag, 2021).

Moreover, students with different personality traits may exhibit varying learning behaviors and performance during the COVID-19 pandemic. Personality can be defined as “the coherent patterning of affect, behavior, cognition, and desires over time and space” (Revelle & Scherer, 2009). Researchers have developed various framework to capture the personality traits. For example, the well-known big five-factor framework (McCrae & John, 1992) proposed to capture and describe personality traits in five dimensions—openness, conscientiousness, agreeableness, extraversion, neuroticism. Personality traits have been demonstrated as one of influential factors in education learning (Chamorro-Premuzic Furnham, 2003; Conard, 2006). Researchers have also discovered the psychological effects by personality traits, such as academic anxiety and declining interest, in the context of educational learning during the COVID-19 pandemic.

**Fig. 1:**
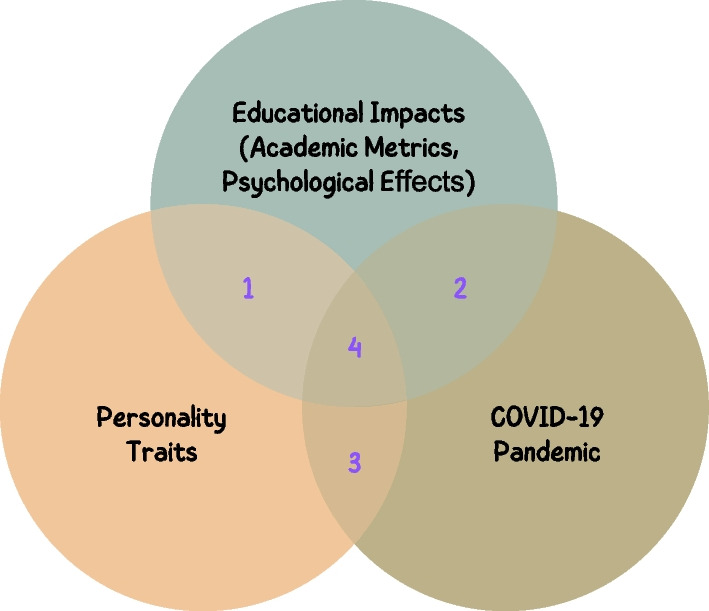
Overlaps in related research

The related research among personality traits, COVID-19 and their educational impacts can be depicted by Fig. 1. In the following discussions, we only focused on the *academic metrics* and *psychological effects* in education as the educational impacts. In this paper, *academic metrics* are defined as quantifiable indicators used to reflect and assess students’ learning behaviors and performance. These metrics may include, but are not limited to, resource usage, class attendance, frequency of engagements, behaviors related to assignment submissions (such as late submissions and the number of attempts), academic performance on quizzes or exams, and others. The *psychological effects* in education refer to the mental or psychological impact that educational experiences and environments can have on students, such as academic anxiety (Hooda & Saini, 2017), student satisfaction (Wiers-Jenssen, 2002), learning interests (Krapp, 1999), and so forth.

These related research can be summarized as follows:

1. *Educational Impacts by Personality Traits*, where studies have looked into how personality traits influence educational outcomes. For example, Zhang et al. found that students with higher levels of agreeableness are more engaged and participate more in online discussions (Zhang, 2020). Ariani found that extroversion positively impacts student communication and engagement (Ariani, 2015). Steinberger et al. discovered that students with lower levels of openness, conscientiousness, agreeableness, and emotional stability were more likely to experience higher levels of academic anxiety (Steinberger, 2021).
2. *Educational Impacts by COVID-19 Pandemic*, where researchers have explored how the COVID-19 pandemic has affected education. Pereira et al. found that the usage of online learning resources increased significantly during the first wave of COVID-19 and continued to increase afterwards (Pereira & Guerreiro, 2021). Feng et al. reported a decrease in learning performance due to the COVID-19 pandemic in China (Feng, 2021). Rashid et al. reported that classroom anxiety even increased in the post-lockdown period due to preventive policies when schools reopened, such as wearing masks, sanitizing hands, and maintaining social distancing (Rashid, 2022).
3. *Impacts by Personality Traits during COVID-19 Pandemic*, where different behaviors, outcomes, and psychological responses have been observed among individuals with various personality traits in various aspects of life, including education. For instance, there are several findings in our daily life, rather than being specific to education only. Individuals with a higher degree of agreeableness may be more prone to experience worry and feelings of loss (Getzmann, 2021). On the other hand, those with more adaptive personality traits tend to have lower stress levels, better sleep quality, and more engagement in household activities (Ahmed, 2021).”
4. *Educational Impacts by Personality Traits Over Pandemics*, where researchers have focused on the educational impact of the COVID-19 pandemic on different personality types among educational stakeholders, such as parents and students. Many studies have explored the impact of personality traits on remote learning during the pandemic. For instance, Morfaki et al. conducted a comprehensive review of the impact of personality traits on remote learning during the COVID-19 pandemic (Morfaki & Skotis, 2022). Yu found that students with strong personality traits, such as agreeableness, conscientiousness, and openness, performed better than those with strong extraversion and neuroticism (Yu, 2021). Quigley et al. found that conscientiousness was a positive predictor of online engagement, and extraversion positively predicted students’ participation and performance in their online studies during the COVID-19 pandemic. However, there are very few studies that made the comparisons among the different lockdown periods. Wen et al. compared the impact of different personality traits on parents’ stress between pre and during pandemic lockdowns and found that positive traits, such as agreeableness, had a protective effect on stress, while negative traits, such as neuroticism, were risk factors (Wen, 2022).

In this paper, we investigate the relationship between academic metrics and personality traits based on a comparison over different COVID-19 lockdown periods. Most existing research in this area has focused on psychological effects such as academic anxiety or learning satisfaction. However, there has been limited research comparing academic metrics among students with different personality traits, particularly across different lockdown periods. Our study aims to fill this gap by exploring the relationship between academic metrics and personality traits through a comparison of pre-lockdown, during-lockdown, and post-lockdown periods. However, due to limitations in our data, our analysis is limited to two types of academic metrics: students’ behaviors in assignment submissions (such as late submissions and the number of attempts) and their final course grades.

The remaining of the paper is organized as follows. We discuss related work in Section “Related work”, and introduce the process of data collection and point out our research problems in Section “Data collection and descriptions”. We discuss our quantitative analysis and comparison results in Section “Exploratory analysis and results”. The summary of the findings and the limitations in our study, as well as the future work are described in Section “Conclusions and practical implications”.

## Related work

In this section, we discuss the related work, especially the overlapped research among personality, education impacts and comparisons among lockdown periods, depicted by Fig. 1.

### Personality traits and collections

Personality traits are a fundamental aspect of individual differences and can have significant impacts on various domains of life, including education. There are multiple frameworks for capturing personality traits, which describe human personalities through different dimensions. Examples of such frameworks include the big five-factor model (FFM) (McCrae & John, 1992), TKI (Thomas, 1992), RIASEC (Holland, 1997), HEXACO (Ashton & Lee, 2007), etc. Personality traits can be collected through the use of well-designed questionnaires, known as personality inventories, associated with each framework.

Take FFM for example, it describes human personalities in terms of five dimensions—Openness, Conscientiousness, Agreeableness, Extraversion, Neuroticism. *Openness* describes an individual’s intellectual curiosity and preference for novelty and variety. *Conscientiousness* refers to one’s level of discipline, organization, and achievement orientation. *Extraversion* reflects the degree of engagement in social activities and the factors of friendliness, gregariousness, and assertiveness. *Agreeableness* measures an individual’s cooperation and trust, as well as modesty, sympathy, and altruism. *Neuroticism* represents the degree of emotional stability. Personality traits in FFM can be measured and collected through questionnaires such as the 44-item Big Five Inventory (John et al., 1999) and the Ten-Item Personality Inventory (TIPI) (Gosling, 2003). In our literature review, most research in educational analysis (Hong, 2021; Sahinidis & Tsaknis, 2021; Steinberger, 2021; Zheng & Subramaniyan, 2021) utilized the FFM framework, while only a limited number of studies utilized other frameworks such as HEXACO, as selected by Volk (2021).

In addition to personality inventory, researchers can also infer the personality traits by using natural language processing techniques. Zhou et al. built conversational agents to serve as AI interviewers who engage a user in a one-on-one, text-based conversation, where the personality traits in FFM can be inferred from users’ textual responses from the conversations with the chatbot (Zhou, 2019).

### Educational impacts by personality traits

Several studies have been conducted to reveal the impacts on educational learning by personality traits. For example, Chamorro et al. reported that conscientiousness positively, extraversion and neuroticism negatively correlated with examination grades (Chamorro-Premuzic & Furnham, 2003). The results by Conard et al. inferred that conscientiousness predicted three academic outcomes (GPA, course performance, and attendance), incrementally over academic ability and other traits (Conard, 2006). Busato et al. also discovered that conscientiousness had a positive correlation with academic success (Busato, 2000).

In addition to the academic performances, researchers also found that personality traits could affect the participation and engagements of the learners. For example, Ariani’s study revealed that extraversion has a positive effect on student communication and engagements (Ariani, 2015). Zhang et al. conducted research and found that students with higher levels of agreeableness are more active and participate more frequently in online discussions (Zhang, 2020). Moreover, personality traits may have direct psychological effects on educational learning. Steinberger et al. discovered that students with lower levels of openness, conscientiousness, agreeableness, and emotional stability are more prone to experience higher levels of academic anxiety (Steinberger, 2021). Cohen et al. found that openness to experience and conscientiousness significantly predicted students’ satisfaction in online education (Cohen & Baruth, 2017).

The usage of personality traits in educational recommender systems (Wu, 2018; Zheng & Subramaniyan, 2021) can also reveal its importance in education. Personality traits can help achieve better personalization in recommender systems, in comparison with regular demographic information (e.g., age, gender, nationality).

### Educational impacts by COVID-19 pandemic

The COVID-19 pandemic has had a profound impact on education globally, affecting students, teachers, and educational systems as a whole. First of all, it gives a rise to the promotion of remote learning, where it also leaves a challenge for ones who face access or technology barriers for both educators and learners (Abuhammad, 2020; Pokhrel & Chhetri, 2021; Rahiem, 2020). Almanar et al. claimed that the compatible devices and high-quality Internet connections became one of the influential factors in educational learning during the pandemics (Almanar, 2020). Moreover, the lack of face-to-face communications and interactions can reduce engagements and learning interests, and further affect the learning outcomes. The findings by Afzal Crawford (2022) show that students’ engagement and self-motivation have collectively moderately explained 35% of the variance in their learning performance.

The COVID-19 pandemic has also had significant impacts on students’ psychological well-being, including academic anxiety (Hooda & Saini, 2017; Kamran & Naeim, 2021; Rashid, 2022) and mental health issues such as feelings of loneliness and isolation from social interactions (Vaillancourt, 2021; Houghton, 2022). In a study by Fawaz and Samaha (2021), it was found that remote learning during the pandemic led to an increase in depression, anxiety, and stress among undergraduate students, with a significant correlation between student satisfaction and the prevalence of these mental health issues. Similarly, (Jehi, 2022) reported that more than one-third of students experienced anxiety during the early stages of the pandemic. Even after schools resumed on-campus learning following the lockdowns, the impacts of academic anxiety were still prevalent (Rashid, 2022; Kamran & Naeim, 2021). One potential reason for this could be the preventive policies that were put in place when schools reopened at the early stage, such as mandatory mask-wearing, hand sanitization, and social distancing (Rashid, 2022).

The variations in the impact of the pandemic on academic performance among individuals can differ based on geographical locations and educational institutions. For instance, Feng et al. observed that the academic performance of primary and secondary students in China worsened significantly after the pandemic, as compared to before (Feng et al., 2021). Conversely, Albeta et al. found no significant differences in the learning performance of 269 chemistry students in Indonesia, when comparing pre and during-pandemic (Albeta et al., 2021). Limniou et al. observed that undergraduate students from a UK university with high level of self-regulation and digital capabilities were able to keep focused and engaged during the lockdown, which results in more first-class grades. Others who preferred the face-to-face teaching delivery process may get bored easily in learning and struggled to work consistently (Limniou, 2021). Cavanaugh et al. investigated the impact on student performance during pandemics from 191 public higher education institutions in USA, and they found that the overall grade point average was even increased by 0.10 (out of 4.0), and they believed that the student grades as a whole did not suffer from the difficulties of learning during pandemics (Cavanaugh, 2023).

### Educational impacts by personality traits over pandemics

Studies have investigated the effects of personality traits on educational outcomes during the COVID-19 pandemic, with a focus on psychological factors such as class satisfaction and academic anxiety. Sahinidis and Tsaknis (2021), Iterbeke and De Witte (2022) found that openness and conscientiousness have a positive correlation with student satisfaction in synchronous online learning, while neuroticism has a negative correlation. Hong et al. also found that higher levels of neuroticism result in more negative emotions during lockdowns, according to results from 273 participants (Hong, 2021). Steinberger et al. discovered negative correlations between openness and several types of anxiety (e.g., class anxiety, interpretation anxiety, fears of asking for help), while extraversion, agreeableness, and conscientiousness only showed negative correlations with limited types of anxiety (Steinberger, 2021).

In contrast, only a limited number of studies have explored the impact of personality traits on academic metrics (e.g., engagement, dishonesty, exam grades, attendance, etc.) during the pandemic, due to difficulties in data collection. Quigley et al. found that conscientiousness has a positive impact on engagement in online learning among 301 undergraduate students (Quigley et al., 2022). Yu et al. found strong correlations between personality traits and online learning outcomes such as assignment scores, sign-ins, video watching progress, and discussions (Yu, 2021). Steinberger et al. examined academic dishonesty using academic misconduct and integrity measures, and they found that statistics anxiety played a role as a mediator between personality traits and academic dishonesty. This suggests that personality traits could contribute to more academic anxiety, leading to increased instances of academic dishonesty (Steinberger et al., 2021).

### Comparisons among pre, during and post-lockdowns

Limited research has been done to compare the effects of personality traits on academic metrics among pre, during, and post-lockdowns. This is likely due to the difficulties in data collection. Our previous studies have compared students’ behaviors in assignment submissions (e.g., late submission, number of attempts) and class grades between pre, during, and post-lockdown periods among graduate students in the United States (Zheng & Zheng, 2022), but there was no analysis of personality traits. Steinberger et al.  (2021) looked at the correlations between personality traits and academic dishonesty during pre and during lockdown periods. Wen et al. investigated the relationship between parents’ personality traits and stress pre and during the pandemic (Wen et al., 2022). Rashid et al. examined classroom anxiety post-lockdown without considering personality traits or comparing with other lockdown periods (Rashid et al., 2022). To the best of our knowledge, there is no existing research that has explored the educational correlations between academic metrics and personality traits through a comparison of pre, during, and post-lockdown periods.

## Data collection and descriptions

In this section, we introduce how we collected the necessary data for analysis. The data used in this paper is a matched data set from two data resources: *ITM-Academics* and *ITM-Rec* data sets.

**Fig. 2:**
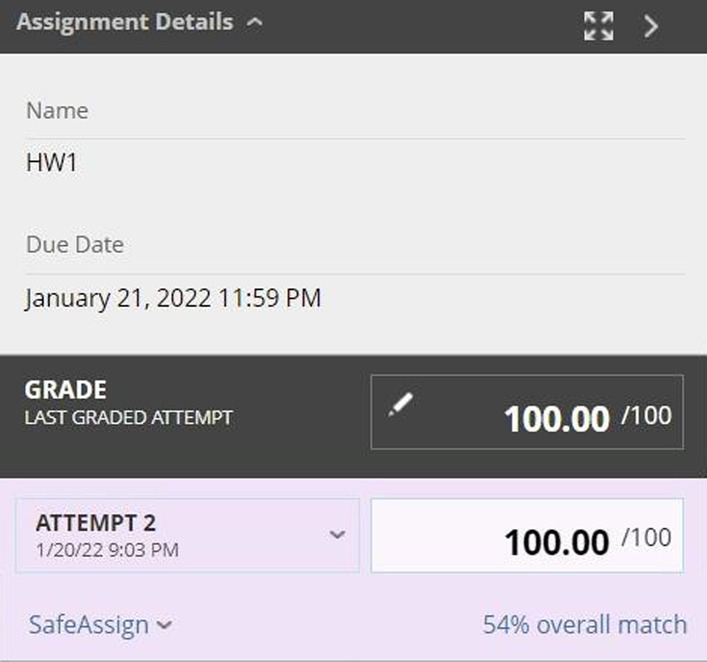
Snapshot on blackboard system

The *ITM-Academics* data set was used in our previous research (Zheng & Zheng, 2022) to examine the differences in academic metrics among pre, during and post-lockdown periods. More specifically, we selected graduate students in the ITM department who enrolled in the data analytics specialization, and collected their academic performance (i.e., course grade) and behaviors in assignment submissions in each course from the Blackboard learning system.^1^ Figure 2 presents an example of the grade details associated with one assignment. We were able to collect two behaviors in assignment submissions on Blackboard—*late submission* (i.e., a binary value with 1 indicating late submissions) and the *number of attempts* a student made in each assignment. The process of data collection was a manual process by the teaching assistants assigned to the class, due to that there are limited API functions offered by the Blackboard system.

Note that there were 8 assignments on average for each course. We calculated the average value of late submission rates, and average number of attempts over all assignments for each student from each course. An example of the data can be observed from Table 1. Three courses from our data science curriculum were involved—data analytics (DA), data science (DS) and artificial intelligence (AI). They were given by a same instructor and had similar level of difficulties.

Our university runs classes by following the semester system in USA. The Spring semester is operated from early January to early May, while the fall semester is running from middle August to early December. This data was collected from the 2018 Spring to 2022 Fall semester. Note that we utilized a larger data in this paper, rather than the original one in our previous study (Zheng & Zheng, 2022), since we collected more data from the Fall semester in 2022.

**Table 1:** Example of data from ITM-Academics

ID	AvgLate	AvgAttempts	Grade	Course	Semester
367	0.22	1.11	84	DA	2018S
368	0	1	88	DS	2019F
$\ldots$	$\ldots$	$\ldots$	$\ldots$	$\ldots$	$\ldots$

The *ITM-Rec* data set (Zheng, 2018, 2023) was collected from 2017 Spring to 2022 Fall based on a user questionnaire deployed on Qualtrics.^2^ This data was originally utilized to collect students’ preferences on Kaggle data sets, so that we can build personality-based recommender systems to suggest Kaggle data sets for students in our data science curriculum. Due to that we only used students’ personality traits from this data, we only introduce the method of collecting personality traits in this data.

More specifically, we considered the personality traits as students’ demographic information, and collected them through the Ten-Item Personality Inventory (TIPI) (Gosling, 2003), along with students’ age and gender. TIPI is a widely used and well-established measure of personality, and it can help obtain the scores associated with the five dimensions of personality traits in FFM. TIPI includes the ten statements that are listed below. Each dimension in FFM is associated with two questions in the following list.

- I see myself as extraverted, enthusiastic.
- I see myself as critical, quarrelsome.
- I see myself as dependable, self-disciplined.
- I see myself as anxious, easily upset.
- I see myself as open to new experiences, complex.
- I see myself as reserved, quiet.
- I see myself as sympathetic, warm.
- I see myself as disorganized, careless.
- I see myself as calm, emotionally stable.
- I see myself as conventional, uncreative.

**Fig. 3:**
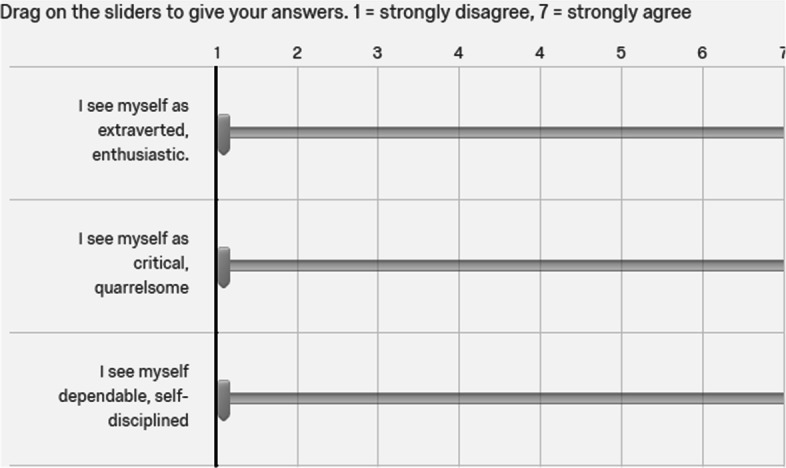
Snapshot of TIPI in Our Questionnaire

Respondents rate each item on a 7-point Likert scale, e.g., 1 (strongly disagree) to 7 (strongly agree), indicating their level of agreement or disagreement with each statement, as shown by Fig. 3. The scores will be eventually aggregated to estimate the degree of the seriousness in five dimensions, including openness (O), conscientiousness (C), extraversion (E), agreeableness (A), neuroticism (N). An example of the data used in this paper can be shown by Table 2.

**Table 2:** Example of data from ITM-Rec

ID	Age	Gender	O	C	E	A	N
367	20–24	M	5	5	6	5	4
368	25–30	F	6	5	4	4	5
$\ldots$			$\ldots$	$\ldots$	$\ldots$	$\ldots$	$\ldots$

The matching process was carried out by using the student ID between two datasets, i.e., an inner join between Table 1 and Table 2, which resulted in a combined dataset with 282 unique students (41% female, 59% male, 37% aged 20 to 24, and 45% aged 25 to 30) and 386 academic records (as seen in Table 1). Keep in mind that a student could have taken multiple courses, thus leading to multiple academic records for that student. The semesters were divided into three phases: pre-lockdown (2018 Spring to 2019 Fall) with on-campus learning, during-lockdown (2020 Spring to 2021 Summer) with remote learning, and post-lockdown (2021 Fall to 2022 Fall) with the return to on-campus learning. Further information about the data can be found in Table 3. The number of students in Table 3 is greater than 282 because some students took classes in multiple semesters (e.g., a student could have taken classes in both 2019F and 2020 S).

**Table 3:** Overview of matched data

	Semester	Course			Number of students	Number of records
		DA	DS	AI		
Pre	2018S	$\times$	$\times$		186	222
	2018F	$\times$	$\times$			
	2019S	$\times$	$\times$			
	2019F	$\times$	$\times$			
During	2020S	$\times$	$\times$		63	91
	2020F	$\times$				
	2021S		$\times$	$\times$		
Post	2021F		$\times$	$\times$	67	73
	2022S		$\times$			
	2022F	$\times$	$\times$			

Each record in our data refers to the combined information from Tables 1 and 2, including the behaviors in assignment submissions (i.e., average rate in late submissions, and average number of attempts in assignment submissions) and class grades associated with a specific student and course, as well as the lockdown period (e.g., pre, during or post) and the personality traits about this student. An example can be observed from Table 4.

**Table 4:** Example of data records in matched data

ID	AvgLate	AvgAttempts	Grade	Course	Lockdowns	O	C	E	A	N
2	0.09	1.18	88.76	DA	PRE	6	6	5	5	5
3	0.00	1.00	91.00	DS	PRE	6	6	5	5	5
4	0.00	1.18	86.82	DA	DUR	5	6	5	5	5
18	0.00	1.36	87.62	DA	POS	5	5	4	5	7
19	0.00	1.00	90.58	DS	POS	5	5	4	5	7
$\ldots$	$\ldots$	$\ldots$	$\ldots$	$\ldots$	$\ldots$	$\ldots$	$\ldots$	$\ldots$	$\ldots$	$\ldots$

## Exploratory analysis and results

In this section, we first point out the research problems and solutions, followed by the discussions of the results from our quantitative analysis.

### Problems and data slices

The primary objective of this study is to examine the impact of two factors, namely, lockdown periods and students’ personality traits, on academic metrics (late submission rate, number of attempts in assignment submissions, and class grades). The differences in academic metrics will be determined through data analysis and the significance of the differences will be assessed through pairwise comparisons using independent-sample statistical tests, as discussed in Sections “Comparisons among lockdown periods” and “Comparisons among student clusters”.

**Fig. 4:**
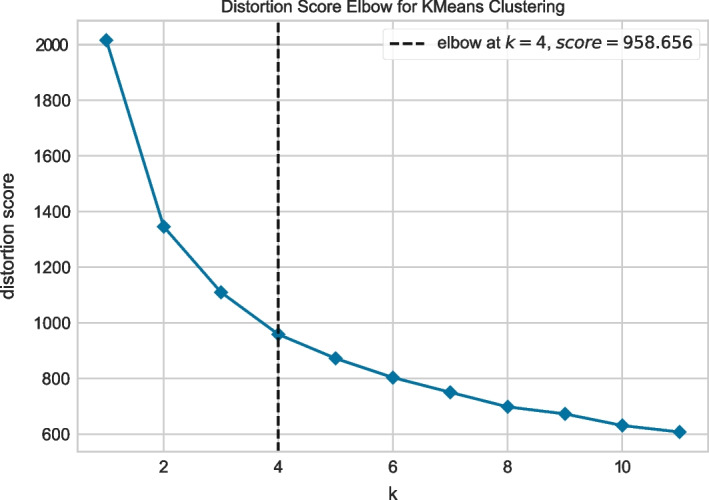
Overlaps in related research

The study aims to examine the relationship between academic metrics and students’ personality traits in the context of different lockdown periods. To do so, the data has been divided into three temporal periods: pre-lockdown, during-lockdown, and post-lockdown. The students’ personality traits were analyzed using K-Means clustering, which resulted in the categorization of students into four clusters. The optimal number of clusters, four, was determined through the use of the elbow method (Fig. 4), as indicated by the distortion score in the K-Means clustering algorithm. The results, shown in Table 5, reveal that students in cluster C$_3$ exhibit higher scores in all personality traits, while those in cluster C$_2$ tend to have lower scores in all traits. Students in clusters C$_1$ and C$_4$ have moderate scores in the personality traits, with C$_1$ having higher scores in agreeableness and neuroticism, and C$_4$ having lower scores in these dimensions and a high score in openness and extraversion.

**Table 5:** Student clusters based on personality traits

	O	C	E	A	N	Number of Students
C$_1$	5.8	5.7	4.6	5.8	5.7	88
C$_2$	4.2	4.2	3.8	4.5	3.5	46
C$_3$	6.7	6.6	6.3	5.9	6.5	78
C$_4$	6.2	5.5	5.7	4.2	4.7	70

Finally, we are able to split the academic records (e.g., class grades and behaviors in assignment submissions) into different slices by *lockdowns* (i.e., pre, during, post) and *student clusters* based on personality (i.e., C$_1$, C$_2$, C$_3$, C$_4$).

Therefore, in this study, two nominal variables were considered: the lockdowns and the student clusters, and three academic metrics were evaluated, namely late submissions, number of attempts, and student grades. The significant differences in the academic metrics were analyzed by considering data subsets for the two nominal variables, using pairwise two-independent sample statistical tests. In particular, the Wilcoxon rank-sum test was employed to evaluate significant differences based on two-sided comparisons, that is, to determine whether there are significant differences between two numerical groups. The Wilcoxon rank-sum test is a widely recognized non-parametric test that does not have any requirements for data normality or equal variances. This test is often used in situations where the normality of the population or population variance is unknown, and it has been utilized in several educational studies (Posner, 2011; Rohe, 2006). Additionally, it is important to note that a 99% confidence level was used in the analysis, and the results were only considered significant if the p-value from the two-independent sample tests was no greater than 0.01.

### Overall analysis

First of all, the statistics of the academic metrics over different groups (i.e., all data, grouped data by lockdown periods, grouped data by student clusters), including mean, median and standard deviation, can be observed from Table 6.

**Table 6:** Statistics of academic metrics

	By lockdown periods				By student clusters			
	ALL	PRE	DUR	POS	C$_1$	C$_2$	C$_3$	C$_4$
Late								
Mean	0.09	0.07	0.09	0.14	0.11	0.09	0.07	0.09
Median	0	0	0	0	0	0	0	0
STD	0.18	0.14	0.20	0.21	0.18	0.19	0.19	0.15
Attempts								
Mean	1.12	1.13	1.10	1.11	1.14	1.05	1.14	1.12
Median	1.0	1.09	1.0	1.0	1.11	1.0	1.0	1.0
STD	0.20	0.18	0.23	0.21	0.17	0.20	0.24	0.18
Grades								
Mean	82.38	83.62	84.01	76.56	81.27	83.46	83.59	81.61
Median	84.34	84.98	84.50	79.20	83.70	85.76	84.57	83.85
STD	9.55	7.99	6.24	14.15	9.46	8.89	7.26	11.96

In order to learn whether *lockdowns* and *student clusters* have significant impacts on three academic metrics, we performed the Kruskal Wallis test which is the non-parametric alternative to the ANOVA technique. The significance results and p-values can be described by Table 7, where *lockdowns* and *clusters* were treated as two nominal factors, and we only present results with a p-value not larger than 0.01.

**Table 7:** p-values in Kruskal–Wallis test

	Factors		
	Lockdowns	Clusters	Lockdowns and clusters
Late	0.01		
Attempts		0.002	0.009
Grades	7.01e-5		0.001

The results shown in the above table indicate that there is a significant relationship between the academic metrics and the two nominal factors, which are the lockdowns and the student clusters derived from their personality traits. It can be observed that students exhibit different patterns of late submissions and grades during the three lockdown periods. Meanwhile, differences in behavior related to the number of attempts in assignment submissions can only be seen among students with varying personality traits. The interplay of lockdowns and students’ personality traits may have an impact on both their grades and their submission attempts.

Furthermore, we employed Pearson correlation to analyze the relationship between the academic metrics and personality traits. The results revealed a negative correlation ($-$ 0.33) between the number of late submissions and grades. This result aligns with expectations, as a high number of late submissions can be indicative of challenges in completing assignments. However, no or weak correlations (i.e., ranging between $-$ 0.1 and 0.1) were observed between the personality traits and academic metrics.

### Comparisons among lockdown periods

**Fig. 5:**
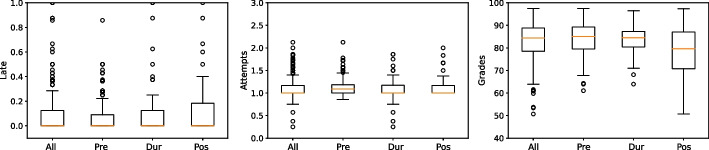
Comparisons among lockdown periods

The statistics of academic metrics across the different lockdown periods can be observed from Table 6. A visual representation of the comparisons can be made through the boxplot in Fig. 5. It is evident that grades in the post-lockdown period were lower than the other periods and there were a higher number of late submissions in the post-lockdown period. To uncover further patterns in these metrics, it is necessary to conduct significance tests.

The results of the Wilcoxon rank-sum test can be shown by Table 8, where we compared academic metrics among different lockdown periods. We present the difference in sample means ($d_1$) and sample medians ($d_2$), as well as p-value of the Wilcoxon rank-sum test (*p*), only if we can identify a significance at 99% confidence level. According to Table 8, it is evident that students demonstrated an increase in late submissions and a decrease in class grades during post-lockdowns in comparison to pre-lockdown periods. Nevertheless, there is no statistically significant difference in the number of late submissions between during- and post-lockdowns. Additionally, students performed worse in post-lockdowns as compared to during-lockdowns. As reported by Rashid (2022), a substantial proportion of students claimed that their concerns and stress levels were higher upon returning to school in post-lockdown periods. This was due to the new protocols of wearing masks, frequent hand sanitization, and social distancing that were put in place during the 2021 Fall and 2022 Spring semesters. This increased level of academic anxiety negatively impacted their academic performances.

**Table 8:** By lockdowns: overall comparisons

	Pre-Dur	Dur-Pos	Pre-Pos
Late			d$_1$ = − 0.07
			d$_2$ = 0
			p = 0.003
Attempts			
Grades		d$_1$ = 7.45	d$_1$ = 7.05
		d$_2$ = 5.30	d$_2$ = 5.78
		p = 2.91e-4	p = 2.69e-5

Table 9 presents comparison of results among different lockdown periods by sliced data associated with each individual student cluster. Namely, we examine significant differences in academic metrics among lockdowns within each student cluster. We did not list the results for the comparison in “Pre-Dur” in Table 9 and the results on cluster C$_2$, since there are no significant differences in these entries.

**Table 9:** Comparisons by using each student cluster

(a) Student Cluster, C$_1$		
	Dur-Pos	Pre-Pos
Late		
Attempts		
Grades	d$_1$ = 5.97	d$_1$ = 5.47
	d$_2$ = 6.07	d$_2$ = 6.57
	p = 0.008	p = 0.005

(b) Student Cluster, C$_3$		
	Dur-Pos	Pre-Pos
Late		d$_1$ = − 0.16
		d$_2$ = − 0.17
		p = 0.002
Attempts		
Grades		

(c) Student Cluster, C$_4$		
	Dur-Pos	Pre-Pos
Late		
Attempts		
Grades	d$_1$ = 16.3	d$_1$ = 15.85
	d$_2$ = 10.55	d$_2$ = 10.18
	p = 0.008	p = 0.001

Most of existing research (Iterbeke & De Witte, 2022; Sahinidis & Tsaknis, 2021; Steinberger, 2021) revealed that neuroticism has a positive relationship with academic anxiety, while openness has a negative one. However, researchers obtained conflicting conclusions in other personality traits due to interindividual differences. The patterns in Table 9 can be summarized as follows:

- Students in cluster C$_3$ exhibit an increase in the number of late submissions in post-lockdowns compared to pre-lockdowns. Conversely, there are no significant differences in late submissions between any two student clusters when analyzed using the entire dataset, as opposed to data sliced based on lockdowns. This trend observed in cluster C$_3$ from Table 9 is linked to the joint impact of both personality traits and lockdown periods. A possible explanation for this is that students in cluster C$_3$ have a relatively higher level of openness and extraversion, leading to more social activities (such as visiting friends, parties, etc.) during post-lockdowns. This, in turn, may result in a higher number of late submissions.
- Furthermore, students in clusters C$_2$ and C$_3$ did not display significant differences in their grades. This result is expected as they are typically high-performing students. This is supported by the statistics presented in Table 6 where it can be observed that students in C$_3$ had the highest mean grades and C$_2$ had the highest median grades, with both being associated with lower standard deviations. The low scores of neuroticisum among students in C$_2$ lead to decreased academic anxiety and no significant differences in academic metrics across the different pandemic periods.
- In comparison, students in C$_1$ and C$_4$ had much poorer academic performances during post-lockdowns. Specifically, students in C$_4$ experienced the largest differences in mean and median grades during post-lockdowns. However, based on the results in Table 9, they did not have significantly worse grades than other clusters. This pattern can again be attributed to the joint impact of personality traits and lockdown periods. The statistics in Table 5 show that students in C$_1$ and C$_4$ have moderate levels of conscientiousness and extraversion. This may have made it more difficult for them to adjust to the changes from during-lockdowns to post-lockdowns in their lives and studies.

### Comparisons among student clusters

**Fig. 6:**
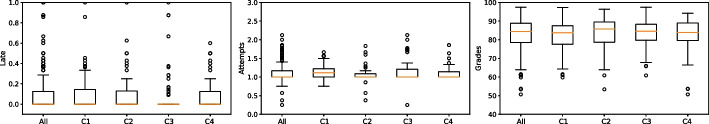
Comparisons among student clusters

The statistics of academic metrics among student clusters can be observed from Table 6. Moreover, a boxplot for comparisons can be depicted by Fig. 6, though the visualizations are not clear to observe the significant differences. To derive solid conclusions, we conducted similar pairwise comparisons based on the Wilcoxon rank-sum test between every two student clusters.

**Table 10:** By clusters: overall comparisons

	Late	Attempts	Grades
C$_1$–C$_2$		d$_1$ = 0.08	
		d$_2$ = 0.11	
		p = 2e-4	
C$_1$–C$_3$			
C$_1$–C$_4$			
C$_2$–C$_3$		d$_1$ = − 0.08	
		d$_2$ = 0	
		p = 0.003	
C$_2$–C$_4$		d$_1$ = − 0.06	
		d$_2$ = 0	
		p = 0.007	
C$_3$–C$_4$			

Table 10 provides results based on the p-values obtained from pairwise comparisons. These findings are consistent with our previous observations in Table 7, indicating that student clusters are only related to the number of attempts made in submitting assignments. In particular, the table reveals that students in cluster C$_2$ tend to make fewer attempts in submitting assignments compared to students in other clusters. This is likely due to their low levels of extraversion and agreeableness. The dimension of agreeableness encompasses behavior related to collaboration, cooperation, teamwork, group discussions, etc. Students with low levels of agreeableness are often resistant to changing their opinions and approach to work. Additionally, students with low levels of extraversion tend to have fewer social activities, such as less teamwork or group discussions, which can result in fewer opportunities to exchange ideas with others and make revisions leading to re-submissions.

In addition, we performed the comparisons associated with each individual lockdown period:

- *Pre-Lockdowns*: Students in C$_1$ have more attempts in comparison with students in $C_2$ (d$_1$ = 0.1, d$_2$ = 0.11, p = 0.002).
- *During-Lockdowns*: Students in C$_1$ have more attempts in comparison with students in $C_2$ (d$_1$ = 0.15, d$_2$ = 0.13, p = 0.005).
- *Post-Lockdowns*: No significant differences identified.

It is not surprising that students in C$_1$ have a higher number of attempts compared to students in C$_2$. However, this pattern was not observed during post-lockdowns, suggesting the impact of both personality traits and lockdowns. Certain conditions during post-lockdowns, such as wearing masks in classrooms and limited in-person office hours, may have influenced their assignment submission behaviors and led to fewer attempts.

## Conclusions and practical implications

In this paper, we explore the educational impacts on academic metrics (e.g., late submission, number of attempts in assignment submissions and class grades) by the two factors – different lockdown periods (e.g., pre, during and post-lockdowns) and student clusters from personality traits. Our findings can be summarized as follows:

- *Overall Pattern*: The results of Table 7 suggest that the grades and late submissions of students are influenced by the lockdown periods, while only the number of attempts in assignment submissions can be affected by the personality traits of different student clusters. The grades are also impacted by both lockdown periods and the characteristics of student clusters.
- *Patterns by Lockdowns*: The students showed an increase in late submissions and a decrease in grades during the post-lockdown period in comparison to the pre- and during-lockdown periods. This could be due to the increased anxiety caused by the school’s policies (such as wearing masks and sanitizing hands) at the start of the post-lockdown period.
- *Patterns by Personality Traits*: Students with lower levels of extraversion and agreeableness submitted fewer assignments, as they may have less collaboration, group discussions, and opportunities to exchange answers with other students
- *Patterns by the Joint Effects*: We observed some intriguing patterns that are influenced by both personality traits and lockdowns. For instance, students with a higher level of openness and extraversion are more likely to engage in social activities such as partying and visiting friends during post-lockdowns, which can lead to increased late submissions.

The following are the limitations of the current study.

- The current study has several limitations in its data sets. Firstly, the data was obtained from only one department at a US institution through a manual process from the Blackboard system, making it challenging to gather data from multiple departments or universities. Secondly, the scope of the academic metrics analyzed is limited. The analysis primarily focuses on behaviors related to assignment submissions, such as the rate of late submissions and the number of attempts, and class grades. Additional dimensions such as attendance and class engagement could provide further insight.
- The available data on post-lockdown is limited to three semesters. During the first two semesters in the post-lockdowns, students were still following preventive measures like wearing masks, sanitizing hands, and maintaining social distancing. As a result, the patterns observed may be different if we can obtain more data from post-lockdown periods when these preventive measures were dropped, e.g., more data from the semesters in 2023 and 2024.
- The current study is lacking user studies, which could have allowed us to gather student perspectives and further understand the identified patterns, such as the decrease in class grades and increase in late submissions in the post-lockdown period.

The outcomes of our research can be applied to generate practical applications or assist educational learning. First of all, the declines in academic performance is worth of being investigated in future. The decrease in academic performance during the post-lockdown period may have two explanations. On one hand, it could be that both teachers and students need to adjust to the transition from remote learning to in-person learning with restrictions like wearing masks and maintaining social distancing. On other hand, the pandemic may have negatively impacted the caliber of students being admitted.

Additionally, our results indicate that the COVID-19 pandemics may have a negative impact on learning behaviors (e.g., increased late submissions) and academic performance (e.g., decreased class grades), and these effects may persist even after the lockdowns have ended. These findings, which are based on comparisons across different lockdown periods, can provide guidance for addressing similar situations or incidents in the future.

The insights gained from the comparison of different personality traits can shed light on the connection between student traits and particular learning behaviors. For instance, students with lower scores in extraversion and agreeableness tend to make fewer attempts in submitting assignments. Those with higher levels of openness and extraversion, on the other hand, are more likely to submit assignments late during post-lockdown periods. These discoveries can aid educators in creating tailored educational plans, such as sending additional email reminders to students who are prone to submitting assignments late.

In future, we plan to observe the effect of post-lockdowns on academic metrics, particularly in 2023 and 2024 when preventive policies are no longer in place in educational institutions. Additionally, we aim to carry out surveys to gather students’ views and understand the impact on these academic metrics, and find ways to assist students in adjusting to learning in a post-lockdown environment.

## Data Availability

The data is not available due to privacy concerns.

## Footnotes

1 https://www.blackboard.com/

2 https://www.qualtrics.com/
